# Prevalence of malnutrition among old age people in Africa

**DOI:** 10.3389/fragi.2022.1002367

**Published:** 2022-11-10

**Authors:** Ahmed Muhye Seid, Netsanet Fentahun Babbel

**Affiliations:** ^1^ School of Public Health, College of Medicine and Health Sciences, Bahir Dar University, Bahir Dar, Ethiopia; ^2^ Department of Public Health, College of Medicine and Health Sciences, Dire Dawa University, Dire Dawa, Ethiopia; ^3^ Department of Nutrition and Dietetics, School of Public Health, College of Medicine and Health Sciences, Bahir Dar University, Bahir Dar, Ethiopia

**Keywords:** Africa, malnutrition, old age people, prevalence, systematic-review, meta-analysis

## Abstract

**Background:** Improved health care and rising life expectancy are creating a growing pool of old age people all over the world, including Africa. Malnutrition in the old age people is associated with both short- and long-term negative health outcomes. However, the reported burdens of malnutrition are fragmented and inconsistent, where more compiled evidence is warranted to aid decision-makers. Hence, this paper is aimed to estimate the pooled prevalence of malnutrition among old age people in Africa.

**Methods:** A systematic search for research reporting the prevalence of malnutrition among old age people (aged above 60 years) was conducted from HINARI/PubMed and Google Scholar databases using combination keywords. Published articles in English language starting from January 2000 to October 2021 were screened. We presented the results based on the standard for reporting systematic review and meta-analysis of observational studies. A random-effect meta-analysis was done to estimate the prevalence of malnutrition along with the 95% confidence intervals. The publication bias was assessed using the funnel plot.

**Results:** A total of 1,442 studies were retrieved based on the search strategy, where only 36 studies (*n* = 15,266 participants) reported from 11 African countries were included for meta-analysis. The reported prevalence of malnutrition ranges from 2.2 to 77.3% across Africa. Overall, the pooled prevalence of malnutrition was 18% (95% CI: 15-22; I^2^ = 98.1; *p* < 0.001). The prevalence is higher in the Central Africa (3.8%; 95% CI: 3.2-4.4), in the community (3.1%; 95% CI: 2.7-3.7), and among advanced age (3.5%; 95% CI: 2.3-5.4).

**Conclusion:** The prevalence of malnutrition in African old age people is high and differs by setting, assessment tool, and country of residence. Hence, due attention to geriatric nutrition is mandatory, and the need for a valid, reliable, and simple screening tool should be thought of.

## Background

The global demographic structure of the population is changing dramatically, notably in Africa, due to several social and economic milestones (United Nations Department of Economic and Social Affairs Population Division, 2019). These resulted in expanding population, improved life expectancy, and increased population size of older ages (World Health Organization, 2017; United Nations Department of Economic and Social Affairs Population Division, 2019). The absolute number of old age people [60 years and above (World Health Organization, 2002)] is expected to grow more rapidly in the coming decades than in any other part of the world (United Nations Department of Economic and Social Affairs Population Division, 2019).

In Africa, the risk of malnutrition among older people is a major challenge to the health care system and needs special and urgent attention. The usual food production practices, marketing style, and living standards have all contributed to an increase in the use of low-cost, packaged products that are high in fat, energy, and salt and yet low in nutritional quality. More importantly, poor infrastructure and limited resources, combined with conflict and poor access to healthcare services, are factors that contribute to the overwhelming levels of malnutrition and food insecurity on the continent (FAO and ECA, 2018). The lack of policy direction for old age people, combined with prevalent chronic illnesses, adds a significant load to the growing burden of malnutrition (Saka et al., 2019; United Nations and Department of Economic and Social Affairs PD (UNDESAPD), 2016). Due to these and other aggravating factors, the African government’s commitment to ending all forms of malnutrition by 2030 (United Nations and United Nations Sustainable Development, 2019) will be difficult to achieve.

While any age group may suffer from malnutrition, it is most common among old age people due to the changes in physiological, psychosocial, and health characteristics of individuals in this age group (Guisado-Clavero et al., 2018). Furthermore, malnutrition has serious and life-threatening consequences that are known to be the main causes of increased morbidity and mortality among old age people (Rosted et al., 2018). Besides, complications of malnutrition, such as osteoarthritis, osteoporosis, diabetes, cardiovascular disease, and hypertension, inflict a significant social and economic burden on them (Gutzwiller et al., 2018).

Timely diagnosis of malnutrition and risk factors is crucial for public health interventions targeting old age people. In addition, previous studies reported that the prevalence of malnutrition among old age people varies between 1.1% and 72.2% in different settings (Barusepang et al., 2017; Ghimire et al., 2018; Konda et al., 2018; Mardani et al., 2018; Wong et al., 2019), where some of the variations could be attributed to differences in the measurement tools, study settings, and demographic groups that have been studied (Visser et al., 2017; Power et al., 2019). Moreover, none of the previous reviews targeted the old age people in Africa (van der Pols-Vijlbrief et al., 2014; Cereda et al., 2016; Leij-Halfwerk et al., 2019; Wolters et al., 2019), where it is anticipated that there are several causes of malnutrition. However, having concrete evidence on the burden of malnutrition using a more rigorous and systematic manner than individual pocket studies is the first step toward enhancing the old age people’s interventions and policy direction. Thus, this paper aimed to provide comprehensive prevalence of malnutrition among old ages, which could potentially provide context-specific evidence to better inform decision-makers in making informed decisions on addressing malnutrition among old age people for a better quality of life.

## Methods

### Search strategies

Before the beginning of this systematic review, we conducted a systematic search of review papers from the Cochrane Library, the International Prospective Register of Systematic Reviews (PROSPERO), and the Joanna Briggs Institute (JBI), and no review had been conducted on malnutrition among old age people in Africa. Then, relevant publications were searched systematically from HINARI, PubMed, and Google Scholar databases from 01 January 2000 to 16 October 2021. We employed the search through relevant combinations of MeSH terms and related keywords, such as “malnutrition”, “obesity”, “overweight”, “old age people”, “older adults”, “elderly”, and “Africa” as indicated in Supplementary File. Reference lists from relevant studies were also manual-searched. Countries or territories included in Africa were defined according to the United Nations (UN) classification (United Nations, 2003). Studies were imported and checked for duplicates in Mendeley Desktop (Version 1.19.3).

### Eligibility criteria

#### Inclusion criteria

Based on the condition, context, and population (CoCoPop) framework (Munn et al., 2015) we included research publications in English language from 1 January 2000 to 16 October 2021 that reported the prevalence of malnutrition among old age people aged 60 years and up in African countries. Intending to get a relatively up-to-date prevalence estimate and obtain a comparable age and malnutrition definition, we restricted studies conducted starting in 2000. All studies that reported the prevalence and/or incidence of malnutrition among old age people were considered regardless of the study design. When multiple studies reported the prevalence of malnutrition based on the same study, the one with the larger sample size was included.

#### Exclusion criteria

Qualitative studies, study protocols, abstracts, opinions, commentaries, case reports, review papers, and studies on African-Americans who do not live on the African continent were all excluded from the review. Duplicated articles and those reporting malnutrition in post-intervention were excluded. Articles without access to the full text were managed using two strategies. First, when outcome data were not reported, we tried to calculate from other additional information in the publications. If not, second method was to contact the corresponding author *via* email. Contacted authors requested full-text papers until 31 October 2021. However, if both strategies were not possible, the study was removed from the review.

### Outcome measuring tools

Studies that measured malnutrition with any standard and validated tools, including questionnaires such as Patient-Generated Subjective Global Assessment (PG-SGA), Mini Nutritional Assessment (MNA), Subjective Global Assessment tools (SGA), or Nutritional Screening Checklist (NCL), anthropometric [body mass index (BMI), mid-upper arm circumference (MUAC), calf-circumference (CC), etc.], or biochemical indices (serum haemoglobin, albumin, etc.) were considered. We used the reported prevalence of malnutrition or undernutrition based on the standard definition set for each nutritional assessment method.

### Study selection and quality assessment

A two-step process was used to select the relevant publications. First, the scan of the titles and abstracts was conducted for the inclusion of references that meet the eligibility criteria. If there were any uncertainties about the relevance of a study, the entire text was retrieved. Second, the full texts were then used to make a judgement on whether or not the study should be included in the review. The two authors work individually on all aspects of the selection process. Disagreements were settled through conversation.

The selected studies were critically appraised using a ten-item rating checklist designed for research reporting prevalence (Hoy et al., 2012), where better quality articles have a higher score as indicated in Supplementary Table S1.

### Data extraction

Data extraction was performed by two independent authors using a prespecified standard format prepared in Excel from the full-text articles. The extract comprised the following information: general (authors, year of publication, and country), participants (age, sex, and morbidity status), and study details (study design, study setting, sample size, measurement tool used, and outcome measures). Morbidity was coded as one or multi when a single or multiple underlying health conditions were an inclusion criterion.

### Statistical analysis

The pre-coded extracted data in excel format was exported to STATA software version 14 (StataCorp, 2015) for descriptive and meta-analysis. For each study, the unadjusted prevalence of malnutrition and standard errors were calculated, and a logarithmic (log) transformation for proportional data was used to reduce variance and obtain the pooled prevalence as an effect size (ES). Considering the level of methodological heterogeneity and variance within the study, a random effect model of the DerSimonian and Laird (Hanji, 2017) method was used to conduct the meta-analysis.

The heterogeneity between studies was assessed using a forest plot, Cochran’s Q test, and I-square (I^2^) test. The I^2^ values of 25, 50, and 75% with a *p-value* less than 0.10 represent low, medium, and high heterogeneity, respectively (Higgins et al., 2003). Publication bias was checked graphically in a funnel plot supplemented by Egger’s statistics (Shi et al., 2017). We also applied a heterogeneity subgroup analysis, an outlier sensitivity analysis, and meta-regression to identify the potential sources of heterogeneity. An effect size estimate with a *P-value* below 0.05 was considered statistically significant.

## Results

### Search results

**FIGURE 1:**
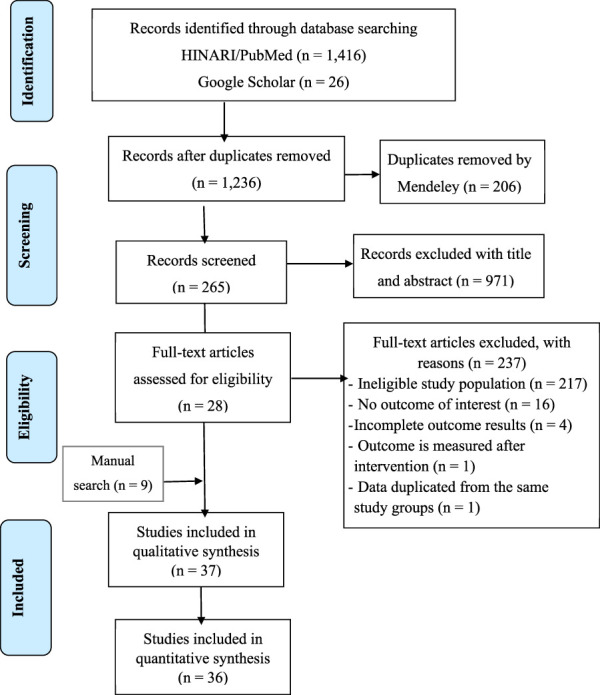
PRISMA flow-chart of studies screened and reviewed.

Atotal of 1,442 articles were identified through searches, of which 206 were removed due to duplicates. With the title and abstract screening, 971 articles were removed depending on the focus of the review (older age in the African continent), relevant nutritional assessment method, and language (other than English). Furthermore, 237 articles were found during the full-text screening. Finally, 37 and 36 articles were selected for qualitative (systematic review) and quantitative analysis (meta-analysis), respectively. Figure 1, presents the comprehensive selection process using the Preferred Reporting Items for Systematic Reviews and Meta-Analyses (PRISMA) statement (Moher et al., 2009).

### Characteristics of included studies

A total of 37 studies reported the prevalence of malnutrition among old age people. While 34 studies (92%) were published within the last 10 years between 2011 and 2020, the remaining 8% were issued between 2001 and 2010. All the included studies were reported from 11 African countries, where more than half (65%, n = 24) were from four countries: Ethiopia, Egypt, Nigeria, and South Africa.

Seven studies (18.9%) were from Ethiopia (Tessfamichael et al., 2014; Hailemariam et al., 2016; Diendéré et al., 2018; Abdu et al., 2019; Adhana et al., 2019; Legesse et al., 2019; Abate et al., 2020) and six studies (16.2%) each from Egypt (Khater and Abouelezz, 2011; Esmayel et al., 2013; Mahfouz et al., 2013; El-sherbiny et al., 2016; El-desouky and Abed, 2017; Allah et al., 2020) and Nigeria (Olasunbo and Olubode, 2006; Adebusoye et al., 2012; Abd-el-gawad et al., 2014; Alao et al., 2015; Adebusoye et al., 2018; Adebusoye et al., 2019). While five studies (13.5%) were from South Africa (Marais et al., 2007; Mkhize et al., 2013; Naidoo et al., 2015; OC et al., 2015; Robb et al., 2017), four studies (10.8%) were from the Central African Republic (CAR) and the Republic of Congo (Andre et al., 2013; Rouvray et al., 2014; Pilleron et al., 2015; Jésus et al., 2017) three studies (8.1%) from Ghana (Aganiba et al., 2015; Agbozo et al., 2018; Apprey et al., 2019), and two studies (6.06%) were from Tanzania (Nyaruhucha et al., 2001; Ijarotimi and Keshinro, 2008).

Except for three studies (two cohorts (Abd-el-gawad et al., 2014; Diendéré et al., 2018) and one case-control (Alao et al., 2015)), all other identified studies were cross-sectional study designs. Malnutrition prevalence was reported in four studies as a comparison between institutional *versus* noninstitutional (Ijarotimi and Keshinro, 2008; Alao et al., 2015), high *versus* low economic classes (Robb et al., 2017), and with and without dementia (Rouvray et al., 2014). However, one study from Tanzania assessed the nutritional status after a post-supplemental food program (Burani and Longo, 2019) and was excluded from the meta-analysis. The majority of the studies (*n* = 22) were conducted in community settings, while 11 studies were conducted among institutionalized elders.

Moreover, a total of 15,266 study participants were found, of whom more than half (58%) were females. The included studies had sample sizes ranging from 40 (Faten et al., 2019) to 2,219 (El-sherbiny et al., 2016) people. Regarding the nutritional assessment tools used, more than half of the studies reported malnutrition based on either the full-or short-form of the Mini Nutritional Assessment tool (MNA-FF: *n* = 15, MNA-SF: *n* = 4). While 18 studies employed various anthropometric measurements such as BMI, MUAC, and CC separately or in combinations to assess malnutrition. One study used an interviewer-administered Nutritional Screening Checklist (NCL) (Esmayel et al., 2013) and another study utilized a combination of MNA and the geriatric nutrition risk index (GNRI) (Faten et al., 2019). Two-thirds (*n* = 23) of the studies reported single (n = 8) or multiple (*n* = 15) underlying clinical conditions such as fracture, heart disease, dementia, Parkinson’s disease, or cancer (Supplementary Table S2).

### Quality of the studies

Based on the quality assessment checklist, studies were either in moderate (6-8 points) (Ijarotimi and Keshinro, 2008; Khater and Abouelezz, 2011; Mahfouz et al., 2013; Abd-el-gawad et al., 2014; Alao et al., 2015; OC et al., 2015; El-desouky and Abed, 2017; Adebusoye et al., 2018; Adebusoye et al., 2019; Andia et al., 2019; Faten et al., 2019; Allah et al., 2020) or high-quality groups (9 or 10 points) (Nyaruhucha et al., 2001; Olasunbo and Olubode, 2006; Marais et al., 2007; Adebusoye et al., 2012; Cheserek et al., 2012; Andre et al., 2013; Esmayel et al., 2013; Mkhize et al., 2013; Rouvray et al., 2014; Tessfamichael et al., 2014; Aganiba et al., 2015; Naidoo et al., 2015; Pilleron et al., 2015; El-sherbiny et al., 2016; Hailemariam et al., 2016; Robb et al., 2017; Agbozo et al., 2018; Diendéré et al., 2018; Abdu et al., 2019; Adhana et al., 2019; Apprey et al., 2019; Legesse et al., 2019; Abate et al., 2020). Study bias was mostly exacerbated as a result of convenience sampling and a low response rate among study participants. In almost all studies, the possibility of external validity was low as the target population was not nationally representative of the country. However, the quality of two studies (Jésus et al., 2017; Burani and Longo, 2019) was not assessed and hence not processed for quantitative data analysis. The first was (Jésus et al., 2017) due to duplication of results from previous data (Rouvray et al., 2014) and the latter (Burani and Longo, 2019) was due to the outcome variable being measured after an intervention (Supplementary Table S3).

### Prevalence of malnutrition

The prevalence of malnutrition varied highly across the studies conducted in different countries, assessment tools used, and healthcare settings. We presented an overview of the findings from various studies using two assessment tools in the section below.

#### Prevalence of malnutrition using BMI

Seventeen studies from hospitals (*n* = 5), the community (*n* = 10), a long-term care center (*n* = 1), and a daycare center (*n* = 1), utilized body mass index (BMI) as a nutritional assessment tool. As low as zero (OC et al., 2015) and 4.0% (Mkhize et al., 2013) of research participants respectively from the old age people daycare center and hospital settings in South Africa were classified as undernourished (BMI ≤18.5 kg/m^2^). Furthermore, the highest prevalence of undernutrition (25.6%) was reported in a Tanzanian hospital (Nyaruhucha et al., 2001). From the community settings, undernutrition was reported at 9.9% in Ghana (Apprey et al., 2019) and 26.4% in the East Africa (Cheserek et al., 2012).

On the other hand, 77% of the old age people studied in a South African hospital (Mkhize et al., 2013) were overweight or obese. None of the study participants in Tanzania were obese or overweight (Nyaruhucha et al., 2001). While 15.3% of study participants in the East Africa (Cheserek et al., 2012) and 42.1% in Ghana (Apprey et al., 2019) were affected by overweight or obesity in the community settings.

#### Prevalence of malnutrition using MNA

Old age people in the community were more susceptible to malnutrition when using MNA compared to hospitals and long-term care centers. The prevalence ranged from 5.7% in Niger (Andia et al., 2019) to 56% in Egypt (Esmayel et al., 2013) and 18% in Egypt (Esmayel et al., 2013) to 58.5% in Niger (Andia et al., 2019), respectively. While 2.24% (Adebusoye et al., 2018) to 40.46% (Abd-el-gawad et al., 2014) and 11.8% (Adebusoye et al., 2012) to 52.7% (Abd-el-gawad et al., 2014) of old age people all from the Nigerian hospitals were malnourished and at risk of malnutrition, respectively. Besides, 7.3% in South Africa (Robb et al., 2017) and 10.8% in Egypt (Khater and Abouelezz, 2011) were malnourished using MNA from the long-term care center.

Similarly, 10.9% of community study participants in Egypt (El-sherbiny et al., 2016) and 77.3% in Ethiopia (Adhana et al., 2019) were malnourished, while none from Ethiopia (Adhana et al., 2019) and 41.9% from Egypt (El-sherbiny et al., 2016) were at risk of malnutrition using MNA-SF. Moreover, 4.57% of hospital study participants in Nigeria (Adebusoye et al., 2019) were malnourished, while 95.43% were either normal or at risk of malnutrition.

### Meta-analysis

**FIGURE 2:**
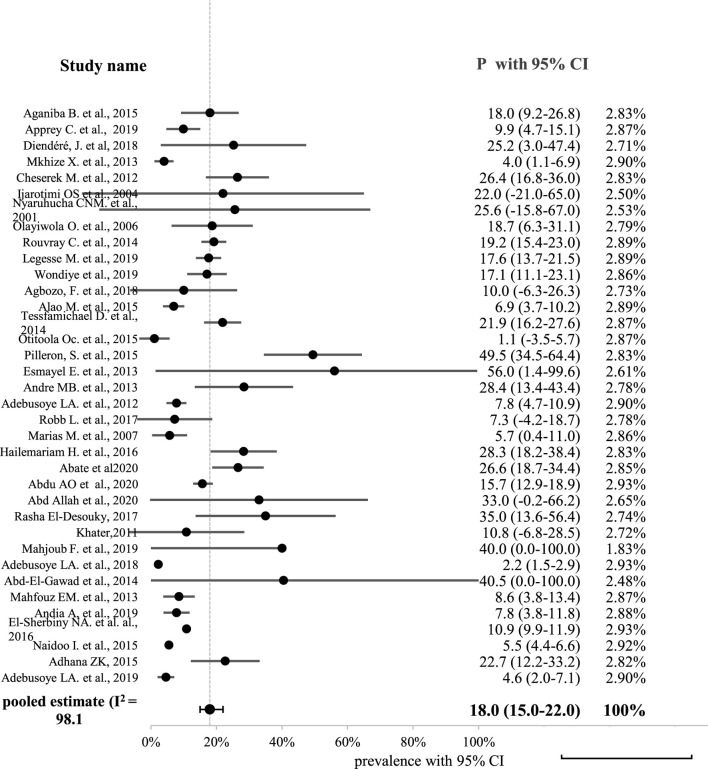
Forest plot displaying the pooled prevalence of malnutrition among old age people in Africa.

Sufficient data were available to conduct pooled malnutrition prevalence estimates for two nutrition screening tools: BMI (n = 16) and MNA (n = 20). Since the full-and short-form of MNA and NCL tools are similar in the interpretation of the outcome variable, they were treated as similar tools. The random pooled prevalence of malnutrition in Africa was 18% (95% CI: 15-22; *p* < 0.001) (Figure 2).

As illustrated in Figure 2, a high level of statistical heterogeneity was observed between studies (heterogeneity of Cochran Q statistics = 1828.98 with *df* = 35 and *p-value* < 0.001; I^2^ = 98.1%; *Tau-square* (τ^2^ = 0.01).

**FIGURE 3:**
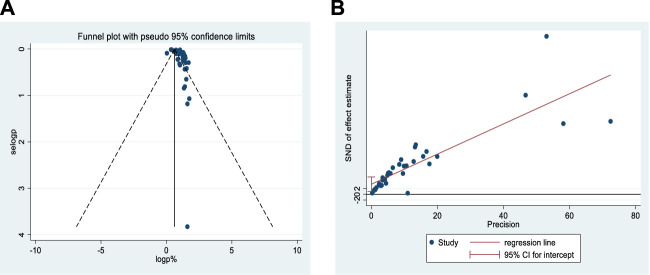
Probability of publication bias for included studies **(A)** funnel plot and **(B)** Egger’s publication bias plot.

Moreover, a minimal publication bias has been noted in the published studies. Studies with lower standard error and larger sample size might be included, as indicated in of the funnel and Egger’s publication plots (Figure 3). Smaller studies with larger standard errors plotted on the right of the funnel plot tend to have both smaller and larger odds ratios. This may indicate the existence of bias due to small-study effects. Moreover, the Egger plot shows that the data near the origin are unsystematically elevated, where the confidence interval (CI) does not include zero, indicating asymmetry in the funnel plot and evidence of publication bias.

Furthermore, Egger’s regression asymmetry test value was 3.50 (*p* = 0.008), suggesting publication bias. Here, the positive coefficient indicates that small studies overestimate the effect size. However, no study appeared to have a significant impact on the summary effect size in the sensitivity analysis, strongly suggesting that the effect estimate is consistent across groups and potentially giving a reliable estimate of the outcome.

### Subgroup analysis and meta-regression

Subgroup analysis and meta-regression were performed for the study regions, settings, assessment tools, age group, and presence of multimorbidity using the random-effects inverse-variance model with the DerSimonian-Laird estimate. In general, a relatively high prevalence of malnutrition was observed in central Africa (3.76%; 95% CI: 3.22-4.39) as compared to other regions of the continent. Similarly, the community old age people (3.12%; 95% CI: 2.66-3.67), older age groups (3.54%; 95% CI: 2.32–5.41), and those with no reported clinical condition (3.16%; 95CI: 2.62–3.80) had a higher malnutrition burden than their counterparts. The prevalence estimates showed a statistically significant variation among all subgroups except for the assessment tools used (*p* = 0.430) (Table 1).

**TABLE 1:** Subgroup analysis for the prevalence of malnutrition among old age people, 2021.

Subgroup	No of studies included	Prevalence with 95%CI	Cochran’s Q statistics for heterogeneity	P value	I2
Regions					
Northern Africa	8	2.82 [2.72–2.92]	3.93	0.788	0.0%
Western Africa	10	2.46 [1.95–3.11]	263.06	<0.001	96.6%
Central Africa	3	3.76 [3.22–4.39]	2.08	0.354	3.7%
Eastern Africa	10	3.46 [2.29–5.22]	312.49	<0.001	97.1%
Southern Africa	5	1.79 [1.41–2.26]	60.54	<0.001	93.4%
Between groups			42.08	<0.001	
Study settings					
Community	21	3.12 [2.66-3.67]	829.56	<0.001	97.6%
Hospital	11	2.12 [1.67–2.70]	132.88	<0.001	92.5%
Long-Term Care	3	2.34 [2.08–2.63]	0.31	0.859	0.0%
Elderly day care center	1	1.04 [0.87–1.24]	0.00	-	-
Between groups			86.79	<0.001	
Assessment Tools					
BMI	16	2.98 [2.37–3.75]	213.68	<0.001	93.0%
NCL	1	5.74 [0.71–46.74]	-	-	-
MNA	15	2.45 [2.11–2.85]	264.09	<0.001	94.7%
MNA-SF	4	2.45 [1.96–3.06]	136.91	<0.001	97.8%
Between groups			2.76	0.430	
Age group					
≥60	26	2.54 [2.17–2.98]	1102.79	<0.001	97.7%
≥65	10	3.54 [2.32–5.41]	361.43	<0.001	97.5%
Between groups			2.06	<0.001	
Morbidity					
One	7	1.66 [1.46–1.89]	91.58	<0.001	93.4%
Multi	16	2.84 [2.50–3.22]	205.87	<0.001	92.7%
Not reported	13	3.16 [2.62–3.80]	36.06	<0.001	66.7%
Between groups			16.44	<0.001	
Overall	37	2.77 (2.43–3.16)	1573.48	< 0.001	97.7%

However, only the study setting was statistically significant (adjusted R-squared = 39.2%, *p* = 0.049) predicting the prevalence of malnutrition in the meta-regression analysis.

## Discussion

We conducted this review paper with the intention of estimating the pooled prevalence of malnutrition disaggregated by relevant factors such as study setting and screening tool. According to the findings, the prevalence of malnutrition ranges from 4.0% (Mkhize et al., 2013) to 77.3% (Adhana et al., 2019) across countries, depending on the assessment tools used and the study settings. These prevalence figures are consistent with previous European estimates of 3.8–67.4% (Leij-Halfwerk et al., 2019; Wolters et al., 2019) among older people despite differences in the settings. In contrast, the highest risk of malnutrition was observed in the community-dwelling old age people assessed by the MNA (both full and short forms) than in other studies; 7.5%–77.3% *versus* 5% (Cereda et al., 2016) to 19% (Verlaan et al., 2017). The variation may be due to the economic variables of the countries.

Overall undernutrition was 17.1% in the African continent using BMI, which is comparable to European countries, 3.8–18.2% (Wolters et al., 2019). While 27% of the study participants in the continent were overweight or obese. Furthermore, many studies (*n* = 14) in our review used BMI as a nutritional assessment tool for old age people. Similar studies in Africa still preferred to use BMI for old age people where 19.9% and 30% of subjects were undernourished, overweight or obese, respectively (Mabiama et al., 2021).

However, the BMI cutoff point used to identify underweight can have a significant impact on prevalence estimates, which tend to misclassify the old age people with physical spinal deformities and other factors that can affect height measurements (Elia and Stratton, 2012). Due to such measurement errors encountered, the assessment of nutritional status in the old age people become a challenging task, which warrants more comprehensive and multidimensional assessments including clinical examination, anthropometric measures, laboratory tests, dietary surveys, and social aspects (Ahmed and Haboubi, 2010; Bharadwaj et al., 2016). This means one assessment tool may not be suitable for all settings, which need contextualization (Cereda et al., 2016). Though more than 22 best-validated nutrition screening tools were identified for older adults (Leij-Halfwerk et al., 2019; Power et al., 2019), there has long been a lack of consensus regarding the criteria needed to make a diagnosis of malnutrition.

On the other hand, the meta-analysis revealed a pooled prevalence of malnutrition of 18% (95% CI: 15-22; *p* < 0.001). This is in the range of the ones reported from the world, ranging from 0.8 to 24.6% (Crichton et al., 2018) depending on the study regions, settings, and assessment tools used. The increasing number of an ageing population in Africa and nutritional transitions might have contributed to a higher burden of malnutrition. Our estimate is also consistent with the pooled prevalence of Indian studies (18.29%; 95% CI: 15.24-21.57) (Kushwaha et al., 2020). However, the result is slightly higher than the prevalence of malnutrition reported from the Central Demographic Republic of Congo and Nigeria (14.5%; 95% CI: 0.0-40.4%) (Crichton et al., 2018). This discrepancy could be explained by the use of various assessment procedures and the merging of pooled estimates from two countries where the burden of malnutrition might be higher.

Surprisingly, the current prevalence estimate is lower than the reviews reported from European older persons (48.4%; 95% CI: 41.5-51.8) (Leij-Halfwerk et al., 2019). The disparity could be attributed to economic factors, health literacy, and healthcare access, and the majority of the previous review data came from hospital settings, implying that individuals with higher health risks were included. The differences may also be caused by the publication bias of data pertaining to the African continent.

Still, there was significantly high heterogeneity between the studies (Q = 1873.99; df = 36; *p* < 0.001; T^2^ = 0.01; I^2^ = 98.1%). Sensitivity analysis did not improve this heterogeneity, which is consistent with other previous findings (Cereda et al., 2016; Crichton et al., 2018). Because of the highly complex physiological, social, and temporal nature of malnutrition, which differs from person to person, as well as bias introduced by research design, analysis of the pooled prevalence of malnutrition is difficult to account for all sources of variation (Marshall et al., 2018).

In the subgroup analysis, the prevalence of malnutrition showed a great variation depending on the study regions, study settings, age groups, and clinical conditions of study participants. Malnutrition was found to be prevalent in central Africa (3.76%), in older age groups (3.54%), with no documented clinical condition (3.16%), and in community-dwellers (3.12%). The prevalence was statistically significant in all subgroups except between the assessment tools used (*p* = 0.430). These findings are consistent with the reviews from India (Kushwaha et al., 2020) and the rest of the world (Crichton et al., 2018). While the meta-regression identified the study setting as a statistically significant (adjusted R^2^ = 39.20%, *p* = 0.049) predictor of malnutrition prevalence. This is, however, different from the reviews conducted in India (Kushwaha et al., 2020) and the world (Crichton et al., 2018) where the study region (R^2^ = 27.1%, *p* = 0.026) and assessment tools (R^2^ = 76.47%, *p* < 0.001) were statistically significant predictors of the malnutrition prevalence, respectively. The variation might be due to the difference in assessment tools used between the studies.

### Strength and limitation

To our knowledge, this is the first systemic review and meta-analysis that provides a comprehensive estimate of the prevalence of malnutrition among old age people in Africa, including more recent studies conducted in the continent. Even though this review has its strengths, it is not without limitations. Due to the inherent heterogeneity among the included studies (I^2^ = 98.08%), the interpretation of the current study needs precaution. Being limited to including only English literature and not including research published in books might limit the generalizability of the estimate.

## Conclusion

This systematic review and meta-analysis showed high malnutrition prevalence among old age people which warrants a targeted policy intervention. The prevalence varied greatly depending on the study setting, the tool used, and the country of residence. To better monitor and evaluate, the use of a more reliable, valid, and standardized malnutrition assessment tool is strongly recommended.

## Supplementary material

Supplementary Table S1 Quality appraisal checklist for prevalence studies.

Supplementary Table S2 Characteristics of Selected Studies Using Different Nutritional Assessment Tools.

Supplementary Table S3 Summary of the quality of included studies based on Hoy et al. (24).

Supplementary File Search items for HINARI/PubMed.

## Abbreviations

BMI, Body Mass Index; CC, Calf Circumference; HINARI, Health InterNetwork Access to Research Initiative; MeSH, Medical Subject Headings; MNA, Mini Nutritional Assessment.
