# Supplementary material for: Prevalence of malnutrition among old age people in Africa
Source: Front Aging. 2022 Nov 10;3:1002367. doi: 10.3389/fragi.2022.1002367 (PMC9686835; doi:10.3389/fragi.2022.1002367)
Supplement: Supplementary file 1 [file Table1.docx]

## Supplementary Table 1: Quality appraisal checklist for prevalence studies

| **Items** | | **Quality score** |
| --- | --- | --- |
|  | **External validity Criteria** |  |
| 1. | Was the study’s target population a close representation of the national population in relation to relevant variables? | (1 point) |
| 2. | Was the sampling frame a true or close representation of the target population? | (1 point) |
| 3. | Was some form of random selection used to select the sample, OR was census undertaken? | (1 point) |
| 4. | Was the likelihood of nonresponse bias minimal? | (1 point) |
|  |  | Total (4 points) |
|  | **Internal validity Criteria** |  |
| 5. | Were data collected directly from the subjects (as opposed to a proxy)? | (1 point) |
| 6. | Was an acceptable case definition used in the study? | (1 point) |
| 7. | Was the study instrument that measured the parameter of interest shown to have validity and reliability? | (1 point) |
| 8. | Was the same mode of data collection used for all subjects? | (1 point) |
| 9. | Was the length of the shortest prevalence period for the parameter of interest appropriate? | (1 point) |
| 10. | Were the numerator(s) and denominator(s) for the parameter of interest appropriate? | (1 point) |
|  |  | Total (6 points) |
|  | Overall total | 10 points |

**Methodological quality**

High quality: score > 8
Moderate quality: score 6-8
Low quality: score 0-5

(Source: Hoy et al.(24))
