# Supplementary material for: Prevalence of malnutrition among old age people in Africa
Source: Front Aging. 2022 Nov 10;3:1002367. doi: 10.3389/fragi.2022.1002367 (PMC9686835; doi:10.3389/fragi.2022.1002367)
Supplement: Supplementary file 2 [file Presentation1.pdf]

Supplementary File

Search items for HINARI/PubMed

#1= "Prevalence"[MeSH] OR "Epidemiology"[MeSH]

#2= "Malnutrition"[MeSH] OR "Obesity"[MeSH]

#3= malnutrition [tiab] OR nutrition*[tiab] OR obesity [tiab]

#4= aged [MeSH] OR elder [MeSH]

#5= old*[tiab] OR aged [tiab] OR elder [tiab] OR geriatric [tiab]

#6= Africa [MeSH] OR Africa [tiab]

#7= Angola[tiab] OR Benin[tiab] OR Botswana[tiab] OR Burkina Faso[tiab] OR Burundi[tiab] OR Cameroon[tiab] OR Cape Verde[tiab] OR Central African Republic[tiab] OR Chad[tiab] OR Comoros[tiab] OR Congo[tiab] OR Côte d'Ivoire[tiab] OR Democratic Republic of the Congo[tiab] OR Djibouti[tiab] OR Equatorial Guinea[tiab] OR Eritrea[tiab] OR Ethiopia[tiab] OR Gabon[tiab] OR Gambia[tiab] OR Ghana[tiab] OR Guinea[tiab] OR Guinea-Bissau[tiab] OR Kenya[tiab] OR Lesotho[tiab] OR Liberia[tiab] OR Madagascar[tiab] OR Malawi[tiab] OR Mali[tiab] OR Mauritania[tiab] OR Mauritius[tiab] OR Mayotte[tiab] OR Mozambique[tiab] OR Namibia[tiab] OR Niger[tiab] OR Nigeria[tiab] OR Réunion[tiab] OR Rwanda[tiab] OR Saint Helena[tiab] OR São Tomé and Príncipe[tiab] OR Senegal[tiab] OR Seychelles[tiab] OR Sierra Leone[tiab] OR Somalia[tiab] OR South Africa[tiab] OR Sudan[tiab] OR Swaziland[tiab] OR Togo[tiab] OR Uganda[tiab] OR United Republic of Tanzania[tiab] OR Zambia[tiab] OR Zimbabwe[tiab] OR Algeria[tiab] OR Egypt[tiab] OR Libya[tiab] OR Morocco[tiab] OR Tunisia[tiab]

#8=#2 OR #3

#9= #4 OR #5

#10= #6 OR #7

#11= #1 AND #8 AND #9 AND #10

#12= Filter: date from 2000/01/01 to 2019/12/10; English language; Humans

Combined search terms

((("Prevalence"[MeSH] OR "Epidemiology"[MeSH]) AND ("Malnutrition"[MeSH] OR "Obesity"[MeSH] OR malnutrition [tiab] OR nutrition*[tiab] OR obesity[tiab]) AND (aged[MeSH] OR elder[MeSH] OR old*[tiab] OR aged[tiab] OR elder[tiab] OR geriatric[tiab]) AND (Africa[MeSH] OR Africa[tiab] OR Angola[tiab] OR Benin[tiab] OR Botswana[tiab] OR Burkina Faso[tiab] OR Burundi[tiab] OR Cameroon[tiab] OR Cape Verde[tiab] OR Central

African Republic[tiab] OR Chad[tiab] OR Comoros[tiab] OR Congo[tiab] OR Côte d'Ivoire[tiab] OR Democratic Republic of the Congo[tiab] OR Djibouti[tiab] OR Equatorial Guinea[tiab] OR Eritrea[tiab] OR Ethiopia[tiab] OR Gabon[tiab] OR Gambia[tiab] OR Ghana[tiab] OR Guinea[tiab] OR Guinea-Bissau[tiab] OR Kenya[tiab] OR Lesotho[tiab] OR Liberia[tiab] OR Madagascar[tiab] OR Malawi[tiab] OR Mali[tiab] OR Mauritania[tiab] OR Mauritius[tiab] OR Mayotte[tiab] OR Mozambique[tiab] OR Namibia[tiab] OR Niger[tiab] OR Nigeria[tiab] OR Réunion [tiab] OR Rwanda[tiab] OR Saint Helena[tiab] OR São Tomé and Príncipe[tiab] OR Senegal[tiab] OR Seychelles[tiab] OR Sierra Leone[tiab] OR Somalia[tiab] OR South Africa[tiab] OR Sudan[tiab] OR Swaziland[tiab] OR Togo[tiab] OR Uganda[tiab] OR United Republic of Tanzania[tiab] OR Zambia[tiab] OR Zimbabwe[tiab] OR Algeria[tiab] OR Egypt[tiab] OR Libya[tiab] OR Morocco[tiab] OR Tunisia[tiab])))

In case of Google Scholar advanced search for the topic was as follows:

1. With all of the words: old age people, or elderly
2. With at least one of the words: prevalence, epidemiology, malnutrition, undernutrition, overnutrition, obesity, overweight, old age people, older adult, elderly, Africa, Sub-Saharan Africa, Horn of Africa
3. Where my words occur: in the title of the article
