# Supplementary material for: Prevalence of malnutrition among old age people in Africa
Source: Front Aging. 2022 Nov 10;3:1002367. doi: 10.3389/fragi.2022.1002367 (PMC9686835; doi:10.3389/fragi.2022.1002367)
Supplement: Supplementary file 3 [file Table2.docx]

**Supplementary** **Table 2.** Characteristics of Selected Studies Using Different Nutritional Assessment Tools

| **S/no** | **Authors** | **Country** | **Study design** | **Study setting** | **Sample size** | **Age group** | **Gender** | | **Assessment tool used** | **Nutritional status (outcome variable) in %** | | | | **Multi-morbidity** | |
| --- | --- | --- | --- | --- | --- | --- | --- | --- | --- | --- | --- | --- | --- | --- | --- |
|  |  |  |  |  |  |  | **Male** | **Female** |  | **Under-nutrition** | **At risk of malnutrition** | **Normal- weight/ Nutrition** | **Overweight/ obesity** |  |  |
|  | Aganiba, B. et al., 2015 (62) | Ghana | C/S | Community | 400 | > 65 | 177 | 223 | BMI | 18.0 |  | 60.5 | 21.5 | Not reported | |
|  | Apprey C. et al., 2019 (63) | Ghana | C/S | Community | 375 | > 60 | 127 | 248 | BMI | 9.9 |  | 48 | 30.9/11.2 | Multi | |
|  | Diendéré, J. et al, 2018 (35) | Burkina Faso | Cohort | Hospital | 222 | > 60 | 121 | 101 | BMI | 25.2 |  | 55.0 | 14.9/5.0 | One | |
|  | Mkhize X. et al., 2013 (53) | South Africa | C/S | Hospital | 270 | > 60 | 46 | 224 | BMI | 4.0 |  | 19.0 | 24.0/53.0 | One | |
|  | MJ, C. et al, 2012 (69) | Lake Victoria Basin of East Africa | C/S | Community | 537 | > 60 | 227 | 310 | BMI | 26.4 |  | 58.3 | 10.8/4.5 | Not reported | |
|  | Ijarotimi & Keshinro, 2004 (65) | Tanzania | C/S | Hospital | 100 | > 60 | 51 | 49 | BMI | 22.0 |  | 61.0 | 17.0 | Not reported | |
|  | Nyaruhucha CNM. et al, 2001 (66) | Tanzania | C/S | Hospital | 121 | > 60 | 67 | 54 | BMI | 25.6 |  | 74.4 | 0 | Not reported | |
|  | Olayiwola O.et al, 2006 (47) | Nigeria | C/S | Hospital | 295 | > 60 | 153 | 142 | BMI | 18.7 |  | 60.7 | 15.7/4.9 | Not reported | |
|  | Jésus p. et al., 2017 (60) | CAR & ROC | C/S | Community | 990 | > 65 | 405 | 585 | BMI | 19.2 |  | 52.9 | 19.1/8.8 | Multi | |
|  | Rouvray C. et al., 2014 (61) | CAR & ROC | C/S | Community | 990 | > 65 | 405 | 585 | BMI | 19.2 |  | 52.9 | 19.1/8.8 | Multi | |
|  | Legesse M. et al., 2019 (34) | Ethiopia | C/S | Community | 892 | > 65 | 365 | 527 | BMI | 17.6 |  | 82.4 | | Multi | |
|  | Wondiye K et al., 2019 (35) | Ethiopia | C/S | Community | 554 | > 60 | 295 | 259 | BMI | 17.1 |  | 80.6 | 2.3 | Not reported | |
|  | Agbozo, F. et al., 2018 (64) | Ghana | C/S | Community | 120 | 60-70 | 42 | 78 | BMI | 10.0 |  | 51.7 | 21.7/16.6 | Not reported | |
|  | Alao M. et al., 2015 (48) | Nigeria | Case-control | LTC | 418 | > 60 | 191 | 227 | BMI | 6.9 |  | 53.3 | 24.6/15.1 | Not reported | |
|  | Tessfamichael D. et al., 2014 (40) | Ethiopia | C/S | Community | 757 | > 65 | 194 | 563 | BMI | 21.9 |  | 78.1 | | Multi | |
|  | Otitoola Oc. Et al., 2015 (57) | South Africa | Cohort | Elderly day care center | 46 | > 60 | 8 | 38 | BMI | 0 |  | 18.4 | 80.4 | Not reported | |
|  | Pilleron, S. et al., 2015 (58) | CAR & ROC | C/S | Community | 645 | > 65 | Not  reported | Not reported | BMI | 68.5 |  | 31.5 | | Not reported | |
|  | Esmayel EM. et al, 2013 (42) | Egypt | C/S | Community | 200 | ≥65 | 112 | 88 | NCL | 56.0 | 18.0 | 26.0 |  | Not reported |  |
|  | Andre MB. et al., 2013 (59) | ROC | C/S | Community | 370 | ≥60 | 194 | 176 | MNA | 13.8 | 28.4 | 57.8 |  | Multi |  |
|  | Adebusoye L. A. et al., 2012 (49) | Nigeria | C/S | Hospital | 500 | ≥60 | 176 | 324 | MNA | 7.8 | 11.8 | 80.4 |  | Multi |  |
|  | Robb L. et al., 2017 (54) | South Africa | C/S | LTC | 124 | ≥60 | 35 | 89 | MNA | 7.3 | 55.6 | 37.1 |  | Not reported |  |
|  | El-Sherbiny N.A. et al., al.2016 (41) | Egypt | C/S | Community | 2,219 | ≥60 | 1165 | 1054 | MNA-SF | 10.9 | 41.9 | 47.2 |  | Multi |  |
|  | Naidoo I. et al., 2015 (56) | South Africa | C/S | Community | 984 | ≥60 | 224 | 760 | MNA SF | 51.0 | 5.5 | 43.4 |  | Multi |  |
|  | Adhana, Z K et al., 2015 (37) | Ethiopia | C/S | Community | 423 | ≥60 | 99 | 324 | MNA-SF | 77.3 | 0 | 22.7 |  | Multi |  |
|  | Adebusoye L. et al., 2019 (50) | Nigeria | C/S | Hospital | 350 | >60 | Not reported | Not reported | MNA-SF | 4.57 | 95.43 | |  | Multi |  |
|  | Marias ML. et al., 2007 (55) | South Africa | C/S | Community | 210 | ≥60 | 53 | 157 | MNA | 5.7 | 47.1 | 47.1 |  | Not reported |  |
|  | Hailemariam H. et al., 2016 (36) | Ethiopia | C/S | Community | 548 | ≥60 | 261 | 287 | MNA | 9.3 | 28.3 | 62.4 |  | Not reported |  |
|  | Abate et al., 2020 (38) | Ethiopia | C/S | Community | 662 | ≥65 | 240 | 422 | MNA | 26.6 | 73.4 | |  | One |  |
|  | Abdu AO et al., 2020 (39) | Ethiopia | C/S | Community | 592 | ≥65 | 291 | 301 | MNA | 15.7 | 51.7 | 32.6 |  | One |  |
|  | Abd Allah et al., 2020 (43) | Egypt | C/S | Hospital | 194 | ≥60 | 107 | 87 | MNA | 33.0 | 51.5 | 15.5 |  | Multi |  |
|  | [Rasha El-Desouky](https://pubmed.ncbi.nlm.nih.gov/?term=El-Desouky+R&cauthor_id=30341994) 2017 (44) | Egypt | C/S | Community | 320 | ≥60 | 167 | 153 | MNA | 35.0 | 38.4 | 26.6 |  | Multi |  |
|  | Khater MS et al., 2011 (45) | Egypt | C/S | LTC | 120 | ≥60 | 56 | 64 | MNA | 10.8 | 40.8 | 48.4 |  | Multi |  |
|  | F. Mahjoub et al., 2019 (68) | Tunis | C/S | Hospital | 40 | ≥65 | 21 | 19 | MNA | 40.0 | 52.5 | 7.5 |  | One |  |
|  | Adebusoye, LA. et al., 2018 (52) | Nigeria | C/S | Hospital | 624 | ≥60 | 246 | 378 | MNA | 2.24 | 32.69 | 65.06 |  | One |  |
|  | Abd-El-Gawad et al., 2014 (51) | Nigeria | Cohort | Hospital | 131 | ≥60 | 67 | 64 | MNA | 40.46 | 52.67 | 6.87 |  | Multi |  |
|  | Mahfouz, E.M. et al., 2013 (46) | Egypt | C/S | Community | 350 | ≥60 | 136 | 214 | MNA | 8.6 | 29.7 | 61.7 |  | Multi |  |
|  | Andia A, et al., 2019 (70) | Niger | C/S | Community | 384 | ≥60 | 132 | 252 | MNA | 7.8 | 58.5 | 33.7 |  | Multi |  |

**Abbreviations**: BMI = Body mass index CAR = Central Africa Republic ROC= Republic of Congo

C/S: Cross-section MNA: Mini Nutritional Assessment Tool MUAC: mid-upper arm circumference

LTC: Long term care MNA-SF: Mini Nutritional Assessment Tool- short Form NCL: Nutritional Screening Checklist
