# Supplementary material for: Prevalence of malnutrition among old age people in Africa
Source: Front Aging. 2022 Nov 10;3:1002367. doi: 10.3389/fragi.2022.1002367 (PMC9686835; doi:10.3389/fragi.2022.1002367)
Supplement: Supplementary file 4 [file Table3.docx]

## Supplementary Table 3: Summary of the quality of included studies based on Hoy et al. (24).

| **S/no** | **Author & year of publication** | **Country** | **External validity quality Score** | | | | **Internal validity quality Score** | | | | | | **Total Score** | **Study Quality** |
| --- | --- | --- | --- | --- | --- | --- | --- | --- | --- | --- | --- | --- | --- | --- |
|  |  |  | **1** | **2** | **3** | **4** | **5** | **6** | **7** | **8** | **9** | **10** |  |  |
|  | Aganiba, B. et al., 2015 (58) | Ghana | 0 | 1 | 1 | 1 | 1 | 1 | 1 | 1 | 1 | 1 | 9 | High |
|  | Apprey C. et al., 2019 (59) | Ghana | 0 | 1 | 1 | 1 | 1 | 1 | 1 | 1 | 1 | 1 | 9 | High |
|  | Diendéré, J. et al, 2018 (31) | Burkina Faso | 0 | 1 | 1 | 1 | 1 | 1 | 1 | 1 | 1 | 1 | 9 | High |
|  | Mkhize X. et al., 2013 (49) | South Africa | 0 | 1 | 1 | 1 | 1 | 1 | 1 | 1 | 1 | 1 | 9 | High |
|  | MJ, C. et al, 2012 (66) | Lake Victoria Basin of East Africa | 0 | 1 | 1 | 1 | 1 | 1 | 1 | 1 | 1 | 1 | 9 | High |
|  | Ijarotimi OS & Keshinro OO, 2004 (61) | Tanzania | 0 | 0 | 0 | 1 | 1 | 1 | 1 | 1 | 1 | 1 | 7 | Moderate |
|  | Nyaruhucha CNM. et al, 2001 (62) | Tanzania | 0 | 1 | 1 | 1 | 1 | 1 | 1 | 1 | 1 | 1 | 9 | High |
|  | Olayiwola O.et al, 2006 (43) | Nigria | 0 | 1 | 1 | 1 | 1 | 1 | 1 | 1 | 1 | 1 | 9 | High |
|  | Rouvray C. et al., 2014 (57) | Central Africa (COR & ROC) | 0 | 1 | 1 | 1 | 1 | 1 | 1 | 1 | 1 | 1 | 9 | High |
|  | Legesse M. et al., 2019 (30) | Ethiopia | 0 | 1 | 1 | 1 | 1 | 1 | 1 | 1 | 1 | 1 | 9 | High |
|  | Wondiye K et al., 2019 (31) | Ethiopia | 0 | 1 | 1 | 1 | 1 | 1 | 1 | 1 | 1 | 1 | 9 | High |
|  | Agbozo, F. et al., 2018 (60) | Ghana | 0 | 1 | 1 | 1 | 1 | 1 | 1 | 1 | 1 | 1 | 9 | High |
|  | Alao M. et al., 2015 (44) | Nigeria | 0 | 0 | 0 | 1 | 1 | 1 | 1 | 1 | 1 | 1 | 7 | Moderate |
|  | Tessfamichael, D. et al., 2014 (36) | Ethiopia | 0 | 1 | 1 | 1 | 1 | 1 | 1 | 1 | 1 | 1 | 9 | High |
|  | Otitoola Oc. Et al., 2015 (53) | South Africa | 0 | 0 | 0 | 0 | 1 | 1 | 1 | 1 | 1 | 1 | 6 | Moderate |
|  | Pilleron, S. et al., 2015 (54) | Central Africa (COR & ROC) | 1 | 1 | 1 | 1 | 1 | 1 | 1 | 1 | 1 | 1 | 10 | High |
|  | Esmayel EM. et al, 2013 (38) | Egypt | 0 | 1 | 1 | 1 | 1 | 1 | 1 | 1 | 1 | 1 | 9 | High |
|  | Andre MB. et al., 2013 (55) | Democratic Republic of Congo | 0 | 1 | 1 | 1 | 1 | 1 | 1 | 1 | 1 | 1 | 9 | High |
|  | Adebusoye L.A. et al., 2012 (45) | Nigeria | 0 | 1 | 1 | 1 | 1 | 1 | 1 | 1 | 1 | 1 | 9 | High |
|  | Robb L. et al., 2017 (50) | South Africa | 0 | 1 | 1 | 1 | 1 | 1 | 1 | 1 | 1 | 1 | 9 | High |
|  | El-Sherbiny N.A. et al., al.2016 (37) | Egypt | 0 | 1 | 1 | 1 | 1 | 1 | 1 | 1 | 1 | 1 | 9 | High |
|  | Naidoo I. et al., 2015 (52) | South Africa | 0 | 1 | 1 | 1 | 1 | 1 | 1 | 1 | 1 | 1 | 9 | High |
|  | Adhana, Z K et al., 2015 (33) | Ethiopia | 0 | 1 | 1 | 1 | 1 | 1 | 1 | 1 | 1 | 1 | 9 | High |
|  | Adebusoye L. et al., 2019 (46) |  | 0 | 0 | 1 | 1 | 1 | 1 | 1 | 1 | 1 | 1 | 8 | Moderate |
|  | Marias ML. et al., 2007 (51) | South Africa | 0 | 1 | 1 | 1 | 1 | 1 | 1 | 1 | 1 | 1 | 9 | High |
|  | Hailemariam H. et al., 2016 (32) | Ethiopia | 0 | 1 | 1 | 1 | 1 | 1 | 1 | 1 | 1 | 1 | 9 | High |
|  | Abate et al., 2020 (34) | Ethiopia | 0 | 1 | 1 | 1 | 1 | 1 | 1 | 1 | 1 | 1 | 9 | High |
|  | Abdu AO et al., 2020 (35) | Ethiopia | 0 | 1 | 1 | 1 | 1 | 1 | 1 | 1 | 1 | 1 | 9 | High |
|  | Abd Allah et al., 2020 (39) | Egypt | 0 | 0 | 0 | 0 | 1 | 1 | 1 | 1 | 1 | 1 | 6 | Moderate |
|  | [Rasha El-Desouky](https://pubmed.ncbi.nlm.nih.gov/?term=El-Desouky+R&cauthor_id=30341994) 2017 (40) | Egypt | 0 | 0 | 1 | 1 | 1 | 1 | 1 | 1 | 1 | 1 | 8 | Moderate |
|  | Khater MS et al., 2011 (41) | Egypt | 0 | 0 | 0 | 0 | 1 | 1 | 1 | 1 | 1 | 1 | 6 | Moderate |
|  | F. Mahjoub et al., 2019 (64) | Tunis | 0 | 0 | 0 | 1 | 1 | 1 | 1 | 1 | 1 | 1 | 7 | Moderate |
|  | Adebusoye, LA. et al., 2018 (48) | Nigeria | 0 | 0 | 0 | 0 | 1 | 1 | 1 | 1 | 1 | 1 | 6 | Moderate |
|  | Abd-El-Gawad et al., 2014 (47) | Nigeria | 0 | 0 | 0 | 1 | 1 | 1 | 1 | 1 | 1 | 1 | 7 | Moderate |
|  | Mahfouz, E.M. et al., 2013 (42) | Egypt | 0 | 0 | 0 | 1 | 1 | 1 | 1 | 1 | 1 | 1 | 7 | Moderate |
|  | Andia A, et al., 2019 (65) | Niger | 0 | 0 | 0 | 0 | 1 | 1 | 1 | 1 | 1 | 1 | 6 | Moderate |
